# Current Understanding on Aflatoxin Biosynthesis and Future Perspective in Reducing Aflatoxin Contamination

**DOI:** 10.3390/toxins4111024

**Published:** 2012-10-25

**Authors:** Jiujiang Yu

**Affiliations:** Southern Regional Research Center, Agricultural Research Service, United States Department of Agriculture (USDA/ARS), New Orleans, LA 70112, USA; Email: Jiujiang.yu@ars.usda.gov; Tel.: +1-504-286-4405; Fax: +1-504-286-4419

**Keywords:** aflatoxins, mycotoxins, Aspergillus flavus, gene cluster, gene regulation, biocontrol, food contaminants

## Abstract

Traditional molecular techniques have been used in research in discovering the genes and enzymes that are involved in aflatoxin formation and genetic regulation. We cloned most, if not all, of the aflatoxin pathway genes. A consensus gene cluster for aflatoxin biosynthesis was discovered in 2005. The factors that affect aflatoxin formation have been studied. In this report, the author summarized the current status of research progress and future possibilities that may be used for solving aflatoxin contamination.

## 1. Introduction on *Aspergillus flavus*, Aflatoxins, and the Importance for the Economy

Aspergilli belong to the class of imperfect filamentous fungi. Among the approximately 250 known species, many are producers of beneficial secondary metabolites, such as antibiotics and other pharmaceuticals [1]. For example, *Aspergillus terreus*, produces lovastatin, a potent cholesterol-lowering drug. Other Aspergilli secrete antibiotics (penicillin and cephalosporin), antifungals (griseofulvin), and anti-tumor drugs (terrequinone A) [2,3]. Many uncharacterized compounds are produced by Aspergilli through various metabolic pathways. These compounds include pathway end products and pathway intermediates or shunt metabolites formed along these pathways and may also have beneficial pharmaceutical properties that can be a potential source of new drugs. However, there are many secondary metabolites produced by *Aspergillus* species, however, they are not always beneficial. Some of them are even toxic and/or carcinogenic and called mycotoxins. Mycotoxins are structurely very diverse chemical compounds with diverse toxic effects and a variety of biological activities [4]. 

Within the genus *Aspergillus*, *Aspergillus flavus* is the most important economically and most notorious because it produces aflatoxins. *A. flavus* fungus, one of the most abundant soil-borne molds on earth, is a saprobe that is capable of surviving on many organic nutrient sources like plant debris, animal fodder, cotton, compost piles, dead insects and animal carcasses, stored grains, and even immunocompromised humans and animals [5]. It has the ability to survive temperatures ranging from 12 °C to 48 °C, but the optimal growth temperature ranges from 28 °C to 37 °C. Its ability to grow at relatively high temperatures contributes to its pathogenicity toward humans and other warm-blooded animals. For most of its lifecycle, the fungus exists in the form of mycelium or asexual spores known as conidia. Under adverse conditions such as lack of adequated nutrients or water, the fungal mycelium will transform to resistant structures called sclerotia, which can survive in extremely harsh environmental conditions. The fungus overwinters either as spores, sclerotia, or as mycelium in debris. When conditions become favorable, the sclerotia germinate directly to produce new colonies or conidiophores with conidia [6,7,8]. 

The fungus *A. flavus* is a weak and opportunistic plant pathogen, affecting many agricultural crops such as maize (corn), cotton, groundnuts (peanuts), as well as tree nuts such as Brazil nuts, pecans, pistachio nuts, and walnuts. Preharvest contamination of these crops with aflatoxins is common. *A. flavus* also causes the spoilage of post harvest grains during storage. Because *A. flavus* lacks host specificity [9], it can attack seeds of both monocots and dicots, and seeds produced both above ground (corn) as well as below the ground (peanuts). Under weather conditions favorable for its growth, *A. flavus* can cause ear rot on maize, resulting in significant economic losses to farmers [10,11,12]. 

The *A. flavus* toxins were first identified as the cause of a severe animal poisoning incident in England in 1960 called the Turkey X disease [13,14]. Most *A. flavus* produces aflatoxins B_1_ and B_2_ whereas *Aspergillus parasiticus*, produces aflatoxins B_1_, B_2_, G_1_, and G_2_. These four major aflatoxins are named based on their blue (B) or green (G) fluorescence under ultraviolet light, and their relative mobility by thin-layer chromatography on silica gel. Aflatoxin M_1_ is a hydroxylated derivative metabolized from aflatoxin B_1_ by cows and secreted in milk [15]. In addition to aflatoxins B_1_ and B_2_, *A. flavus* also produces many other mycotoxins such as cyclopiazonic acid, kojic acid, beta-nitropropionic acid, aspertoxin, aflatrem and aspergillic acid [16]. 

Aflatoxin B_1_, among the four major types of aflatoxins, is the most toxic and the most potent carcinogen in humans and animals including nonhuman primates, birds, fish, and rodents. Chronic exposure can result in suppressed immune response, malnutrition, proliferation of the bile duct, centrilobular necrosis and fatty infiltration of the liver, hepatic lesions, and even hepatomas. In animal models, aflatoxin B_1_ is modified into a more toxic and carcinogenic by-product during detoxification by a cytochrome P450 monooxygenase in liver [17,18,19,20]. The epoxide form of aflatoxin binds to guanine residues in DNA, forms guanyl-N7 adducts, and induces mutations. One mutation, a G to T transversion [21,22] at the third base of codon 249, a mutation hot spot of the p53 tumor suppressor gene and is generally believed to be the mechanism for initiating hepatocarcinoma formation [23,24,25,26]. The p53 gene encodes a transcription factor involved in cell cycle regulation. It is commonly mutated in human liver cancers [27]. Aflatoxin B_1_ is also a potential immunosuppressive agent [28]. Chronic low level exposure of growing vertebrates to aflatoxins may enhance their susceptibility to infection and tumorigenesis [28]. Aflatoxin B_1_ (AFB_1_) also affects other organs and tissues, such as the lungs and the entire respiratory system [29]. Human hepatocarcinomas are also associated with hepatitis B virus (HBV) and C virus (HCV) infections [18,30,31]. Together with aflatoxins these viruses significantly increased the risk of hepatoma in hepatitis patients [32,33,34,35]. 

Food and feed contamination by aflatoxins is a significant food safety issue in the developing countries sometimes because of lack of detection, monitoring and regulating measures to safe guard the food supply. It is estimated that approximately 4.5 billion people living in developing countries are chronically exposed to largely uncontrolled amounts of aflatoxin that severely results in changes in immunity and nutrition [36]. Major outbreaks of acute aflatoxicosis from contaminated food in humans have been documented in developing countries [20]. For example, in western India in 1974, 108 persons among 397 people affected, died from aflatoxin poisoning [37]. A more recent incident of aflatoxin poisoning occurred in Kenya in July 2004 leading to the death of 125 people among the 317 reported illnesses due to consumption of aflatoxin contaminated maize (corn) [20,37]. In the Kenia case, the aflatoxins were produced by *A. parvisclerotigenus* instead of *A. flavus*. Acute toxicosis is not the only concern. The world health authorities warn that low doses with long-term dietary exposure to aflatoxins is also a major risk as they can lead to hepatocellular carcinoma [22,24,38,39]. International Agency for Research on Cancer (IARC) has designated aflatoxin as a human liver carcinogen [39,40]. This food poisoning problem is rarely observed in the U.S. in humans but does occasionally occur in animals. The most notable recent case involved the reported death of over 100 dogs in 2006 that had consumed tainted dog feed [41]. 

To minimize potential exposure to aflatoxins, maximum levels of aflatoxins in many commodities have been set at levels below 20 ppb by most countries [15,42,43]. Regulatory guidelines of the U.S. Food and Drug Administration (FDA) specifically prevent the sale of commodities if contamination by aflatoxins exceeds 20 ppb total aflatoxins for interstate commerce of food and feedstuff and 0.5 ppb aflatoxin M_1_ in milk. The European Commission has set the limits on groundnuts subject to further processing at 15 ppb for total aflatoxins and 8 ppb for aflatoxin B_1_, and for nuts and dried fruits subject to further processing at 10 ppb for total aflatoxins and 5 ppb for aflatoxin B_1_. The aflatoxin standards for cereals, dried fruits, and nuts intended for direct human consumption are even more stringent, and the limit for total aflatoxins is 4 ppb and 2 ppb for aflatoxin B_1_ [43].

Due to restrictions limiting the trade of contaminated crops, aflatoxin contamination of agricultural commodities is not only a serious food safety concern [10,14,44,45,46,47,48,49,50,51], but it has significant economic implications for the agricultural industry worldwide.

## 2. Genetics and Molecular Biology of Aflatoxin Biosynthesis

**Figure 1:**
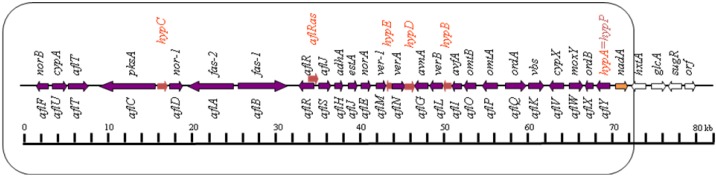
Aflatoxin pathway gene cluster in *A. flavus.* This figure shows the order and location of the 30 aflatoxin pathway genes plus an *aflR* antisense gene clustered together in about 80 kb DNA region. The old gene names are labeled on top of the line and the new gene names sysmatically renamed according to gene convention are labeled below the line [81]. The transcripts of *hypA*, *hypB*, *hypC*, *hypD*, *hypE* and *aflRas* are identified through *Aspergillus flavus* EST. Arrows indicate the direction of gene transcription.

Since the identification of aflatoxins, extensive efforts have been made and expenses incurred worldwide to monitor aflatoxin occurrence and to develop control strategies [52,53,54,55,56,57]. The hallmark discovery of a color mutant that accumulates the brick-red pigment, norsolorinic acid (NOR), in *A. parasiticus* marked a milestone in the understanding the chemistry of aflatoxin biosynthesis [58,59,60,61]. Since NOR is the earliest and the first stable aflatoxin precursor in the aflatoxin biosynthetic pathway [62,63,64], this discovery led to the identification of other key aflatoxin intermediates and established the early step metabolites in the aflatoxin pathway. It provided the opportunity to isolate the first aflatoxin pathway gene that encoding a reductase for the conversion from NOR to eventually aflatoxins [63,65,66]. After the cloning of several important aflatoxin pathway genes, the aflatoxin pathway gene cluster was discovered in *A. parasiticus* and *A. flavus* [67]. The knowledge of the cluster promoted renewed interest in understanding aflatoxin biosynthesis by scientists all over the world. Significant progress has been made in elucidating the biosynthetic pathway, the pathway intermediates, genes, corresponding enzymes, and regulatory mechanisms [44,46,68,69,70,71,72,73,74,75,76,77,78,79,80,81]. As many as 30 genes are potentially involved in aflatoxin biosynthesis (Figure 1). In *A. flavus* and *A. parasiticus* the aflatoxin pathway genes are clustered within a 75-kb region of the fungal genome on chromosome III roughly 80 kb away from telomere [67,81,82,83,84,85,86,87]. 

### 2.1. Conversion of Acetate to Norsolorinic Acid (NOR)

**Figure 2:**
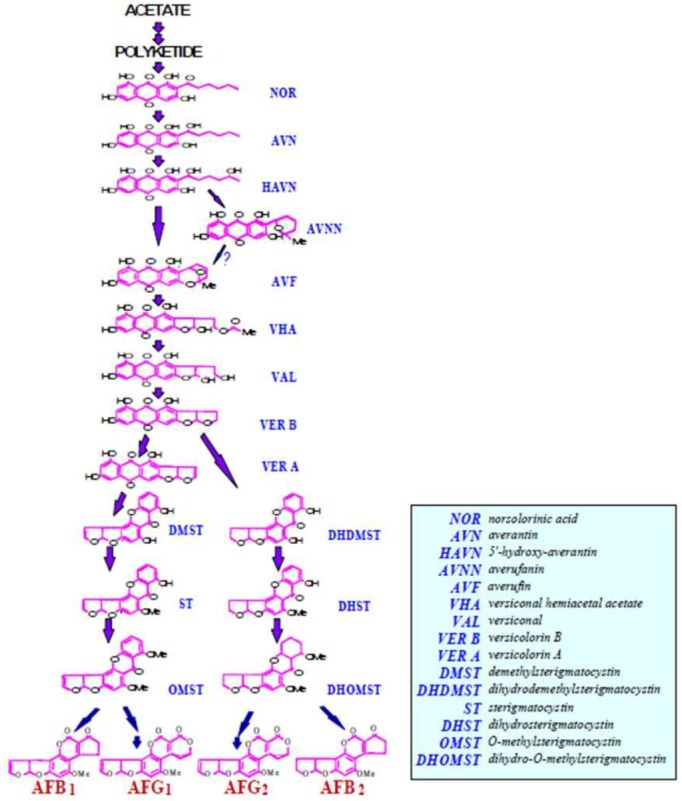
The schematic aflatoxin biosynthetic pathway is presented. The arrows indicate the pathway steps from previous precursor to the next intermediate towards the formation of aflatoxins. The abbreviations are shown on the right.

The first stable aflatoxin precursor was confirmed to be norsolorinic acid (NOR) [59,60,88] (Figure 2). A hexanoyl starter unit is the initial substrate for aflatoxin formation [65]. Two fatty acid synthases (FAS) and a polyketide synthase (NR-PKS, PksA) are involved in the synthesis of the polyketide from a hexanoyl starter unit. Seven iterative, malonyl-derived ketide extensions are required to produce norsolorinic acid anthrone (noranthrone) [76,83,84,85,89,90,91,92,93,94,95,85,89]. Mahanti *et al.* [ 96] cloned, by genetic complementation, a 7.5-kb large transcript which is required for NOR formation in a blocked *A. parasiticus* mutant. Its protein has high degree of similarity (67%) and identity (48%) to the beta-subunit of FASs (FAS1) of *Saccharomyces cerevisiae* and *Yarrowia lipolytica*. Metabolite feeding and gene disruption experiments further confirmed that *uvm8* encodes a subunit of a novel fatty acid synthase (FAS) directly involved in the backbone formation of the polyketide precursor of NOR during aflatoxin biosynthesis, therefore, on the basis of its function, the *uvm8* gene was renamed *fas-1A*. In the revised naming scheme, the *fas-1A* gene was renamed as *fas-1*, it encodes fatty acid synthase-1 in the aflatoxin biosynthetic pathway gene cluster (Figure 1). Another large transcript (*fas-2A*) which encodes an alpha-subunit of fatty acid synthase in the aflatoxin gene cluster was reported [96]. The gene *fas-1A* and *fas-2A* were renamed as *fas-1* and *fas-2*. They encode two fatty acid synthases (FASα and FASβ) [97]. In *A. nidulans* the involvement of FASs in sterigmatocystin (ST) biosynthesis was also confirmed and were named *stcJ* and *stcK* in the ST cluster [90,95]. The biochemical evidence for the role of a fatty acid synthase and a polyketide synthase (PKS) in the biosynthesis of aflatoxin was demonstrated [98]. Further details on the early stage of aflatoxin biosynthesis involving fatty acid synthases and polyketide synthases were reported [89,91,92,99]. The *N*-acetylcysteamine thioester of hexanoic acid was incorporated into NOR in a *fas-1* disrupted transformant. A polyketide synthase gene (*pksA*) in *A. parasiticus* was demonstrated by gene disruption to be required for aflatoxin biosynthesis [73]. The predicted amino acid sequences of these PKSs contain the typical four conserved domains commonly found in other known PKS proteins: β-ketoacyl synthase (KS), acyltransferase (AT), acyl carrier protein (ACP), and thioesterase (TE) [73]. Townsend’s group has dissected the functional domains of the PKS for aflatoxin biosynthesis [76,93,100]. These include domains for the starter unit acyl transferase (SAT) which recognizes hexanoyl CoA and the *N*-acetylcysteamine thioester of hexanoic acid, the acyl carrier protein (ACP), ketosynthase (KS), malonyl-CoA:ACP transacylase (MAT), product template (PT) allowing the iterative steps in forming the polyketide, and a thioesterase/Claisen-like cyclase (TE/CLC) [76]. The predicted product converted by PksA is noranthrone. The conversion of noranthrone to NOR, the first stable intermediate in the pathway [53,60,62,69,101,102,103], is poorly defined, but it has been proposed to be catalyzed by a noranthrone oxidase, a monooxygenase, or to occur spontaneously [64]. Sequence analysis and enzymatic studies support the contention that the *hypC* (a gene in the intergenic region of *pksA* and *nor-1*) gene product is the required noranthrone oxidase involved in the catalysis of the orxidation of norsolorinic acid anthrone to NOR [80] The *fas-1*, *fas-2*, and *pksA* genes were renamed as *aflA*, *aflB*, and *aflC* respectively [81,83,87] (Figure 1). The *aflA*, *aflB* and *aflC* gene homologues in *A. nidulans* are *stcJ*, *stcK*, and *stcA*, respectively [90].

### 2.2. Conversion of Norsolorinic Acid (NOR) to Averantin (AVN)

The first stable aflatoxin (AF) intermediate was identified as NOR produced in *A.* *parasiticus* uv-generated disruption mutants [60,69,103,104] and in *A. flavus* [53,102]. The NOR-accumulating mutants are leaky mutants whose aflatoxin biosynthesis is not completely blocked. By genetic complementation, the gene, *aflD* (*nor-1*), encoding a reductase was cloned [105]. A recombinant Nor-1 protein expressed in *E. coli* catalyzed the reduction of NOR. Therefore, *aflD* (*nor-1*) encodes the ketoreductase needed for the conversion of the 1'-keto group in NOR to the 1'-hydroxyl group of AVN [106]. Disruption of the *aflD* (*nor-1*) gene also confirmed its involvement in conversion of NOR to AVN in aflatoxin biosynthesis [107]. The *aflD* (*nor-1*) homologous gene in *A. nidulans* is *stcE* [90]. Genes homologous to *aflD* (*nor-1*), in the AF cluster, such as *aflE* (*norA*) and *aflF* (*norB*) are predicted to encode short chain aryl alcohol dehydrogenases. These proteins may also be able to catalyze the reduction of NOR to AVN depending on the reductive environment of the cell and may explain the leakiness of the *nor-1* mutation if they are able to complement Nor-1’s function [108].

### 2.3. Conversion of Averantin (AVN) to 5'-Hydroxyaverantin (HAVN)

Radioisotope incorporation experiments provide the earliest evidence in establishing the conversion of AVN to HAVN [109,110]. There are three enzymatic steps that account for the conversion of NOR to averufin (AVF) [111]: (i) NOR to AVN catalyzed by a reductase, (ii) NOR to HAVN catalyzed by a monooxygenase, and (iii) HAVN to AVF catalyzed by a second dehydrogenase. It was also proposed that the oxidation reactions are reversible and that NADPH was the preferred cofactor [112]. The gene previously named *ord-1* encoding a P-450 monooxygenase was cloned and disrupted [113]. Substrate feeding studies of the *ord-1* mutant confirmed that HAVN is the intermediate in the conversion of AVN to AVF. The *ord-1* gene, which has a high degree of sequence similarity to *A. nidulans stcF* [90], was renamed *aflG* (*avnA*).

### 2.4. Conversion of 5'-Hydroxyaverantin (HAVN) to Oxoaverantin (OAVN), and Averufin (AVF)

Averufin is one of the key intermediates in aflatoxin formation [103,114,115,116,117,118,119]. Several intermediates were reported to be involved in the conversion from AVN to AVF [70,118]. One of these is averufanin (AVNN). Studies later demonstrated that it is a shunt metabolite and not a genuine aflatoxin intermediate [94,120]. The cluster gene *aflH* (*adhA*) in *A. parasiticus* was characterized to encode an alcohol dehydrogenase [103,118,121]. It was shown that *adhA* deletion mutants accumulated predominantly HAVN and after prolonged growth, the mutants were able to produce small amounts of AVNN consistant with AVNN being a shunt metabolite. Thus, HAVN might be converted directly to AVF or indirectly to AVF by an additional cytosolic enzyme. Sakuno *et al*. [120] characterized two cytosolic enzymes and a new aflatoxin intermediate named 5'-oxoaverantin (OAVN) as an intermediate between HAVN and AVF. The enzyme for the conversion from HAVN to OAVN is encoded by the *aflH* (*adhA*) gene. The *adhA* gene deletion mutant is leaky indicating that additional enzyme(s) or gene(s) may be involved in the conversion from OAVN to AVF. The enzymatic steps for aflatoxin biosynthesis and the possible involvement of additional enzymes have also been proposed [80,86,122]. The *aflH* (*adhA*) gene in *A. flavus* and the *adhA* gene in *A. parasiticus* share no significant homology at either the DNA or the amino acid level. 

### 2.5. Conversion of Averufin (AVF) to Versiconal Hemiacetal Acetate (VHA)

The conversion of AVF to VHA involves the cytochrome P450 monooxidase, CypX, and another gene, *aflI* (*avfA*). Although *aflI* is required for the conversion, its oxidative role is unclear [123]. *A. nidulans* also has an *aflI* gene homolog (*stcO*) [90,123]. Complementation of an averufin-accumulating mutant, *A. parasiticus* SRRC 165, with the *aflI* gene of *A. flavus* restored the strain’s ability to convert AVF to VHA and to produce aflatoxins [123]. It is likely that the *aflI* (*avfA*) encoded protein along with CypX gene product is involved in the ring-closure step in the formation of hydroxyversicolorone. It is possible that the *avfA* gene product is assocated with the P450 monooxygenase to carry out the conversion as no additional intermediates other that AVF result from the disruption of either gene.

### 2.6. Conversion of Versiconal Hemiacetal Acetate (VHA) to Versiconal (VHOH, Also Abbreviated as VAL)

It has been demonstrated that an esterase is involvement in the conversion of VHA to VHOH (VAL) [57,111,112,124,125,126,127,128]. The esterase was purified in *A. parasiticus* [125,126]. An esterase gene, *aflJ* (*estA*), in the aflatoxin gene cluster was identified [129]. The homologous gene in the *A. nidulans* ST biosynthetic gene cluster is *stcI*. In the *A. parasiticus aflJ* (*estA*) deletion mutants, the accumulated metabolites were mainly VHA and versicolorin A (VERA) [130]. A small amount of versiconol acetate (VOAc) and other downstream aflatoxin intermediates, including VHOH and versicolorin B also accumulated. A metabolic grid containing VHA, VOAc, VHOH, and versiconol (VOH) was previously described and it was suggested that the reactions from VHA to VHOH and from VOAc to VOH are catalyzed by the same esterase [111]. Later, another metabolic grid containing versicolorone (VONE), VOAc, and VHA was identified [131]. Indeed, it has now been proven that the estA-encoded esterase catalyzes the conversion of both VHA to VHOH and VOAc to VOH during aflatoxin biosynthesis [130].

### 2.7. Conversion of Versiconal (VHOH) to Versicolorin B (VER B)

The enzymatic evidence that VHOH is converted toVER B by a cyclase was first provided by Lin and Anderson [132]. This enzyme was identified as versicolorin B synthase and was studied intensively by Townsend’s laboratory [114,133,134,135,136]. The gene was cloned and named *vbs* [114,134,136]. The expected cyclase activity was demonstrated by the expressed recombinant protein of the *vbs* gene [134,135]. The VHOH cyclase [132] and VER B synthase [133] were independently isolated from *A. parasiticus*. The enzyme catalyzes the side chain cyclodehydration of racemic VHA to VER B. This is another key step in aflatoxin formation since it closes the bisfuran ring of aflatoxin, the moiety ultimately responsible for aflatoxin’s toxicity and carcinogenicity. The *vbs* gene was re-named *aflK* (*vbs*) [81]. The homologous gene in the *A. nidulans* ST biosynthetic gene cluster is *stcN*. 

### 2.8. Conversion of Versicolorin B (VER B) to Versicolorin A (VER A)

The critical branch point leading to the formation of either AFB_1_/AFG_1_ or AFB_2_/AFG_2_ is VER B. Similar to AFB_2_/AFG_2_, VER B contains a tetrahydrobisfuran ring and, like AFB_1_/AFG_1,_ VER A contains a dihydrobisfuran ring. The conversion of VER B to VER A requires desaturation of the bisfuran ring of VER B by an unstable microsomal enzyme that requires NADPH [137]. Disruption of *stcL* in *A. nidulans* [138] abolished ST synthesis and resulted in the accumulation of VER B. The *stcL* gene encodes a cytochrome P-450 monooxygenase. The homologue, *aflL* (*verB*), is present in the aflatoxin gene cluster of *A. parasiticus* and *A. flavus* strains. Cultural conditions appear to markedly affect the activity of VER B desaturase and thereby, the final ratio of AFB_1_ to AFB_2_ and AFG_1_ to AFG_2_[94].

### 2.9. Conversion of Versicolorin A (VER A) to Demethylsterigmatocystin (DMST) and Versicolorin B (VER B) to Demethyldihydrosterigmatocystin (DMDHST)

The biochemical conversion steps from VER A to DMST (and VerB to DHDMST) have been described in great detail [139]. The *aflM* (*ver-1*) gene [140], cloned by genetic complementation of VER A-accumulating *A. parasiticus* CS10 (a mutant strain created by knocking out the *verA* gene in aflatoxin-producing strain *A. parasiticus* SU-1), was shown to be responsible for the conversion of VER A to an intermediate that has not been isolated. The *aflM* (*ver-1*) gene was predicted to encode a ketoreductase, similar Nor-1. The *ver-1* homologue, *stcU*, (previously named *verA*) was identified in *A. nidulans* [141]. Double mutation of *stcU* and *stcL* resulted in accumulation of only VER A [141]. The *stcS* gene(previously named *verB*), another cytochrome P-450 monooxygenase gene, was also identified and studies showed that it is also involved in the conversion of VER A to an intermediate in the formation of DMST (possibly the first intermediate, which is then acted upon by Ver-1). Disruption of *stcS* resulted in the accumulation of VER A as did disruption of Ver-1 [142]. Thus, both *stcU* and *stcS* are required for the conversion of VER A to DMST. The *stcS* homologue in *A. parasiticus*, named *aflN* (*verA*), has also been identified [81,87]. A third enzyme is required for the conversion: *hypA* (*aflY*). This gene is predicted to encode a Baeyer-Villiger monooxygenase. Disruption of this gene also led to accumulation of VERA suggesting that, like VER-1, it acts as part of an enzyme complex without allowing the formation of an intermediate. A fourth enzyme, OrdB has also been implicated in the conversion, and like AvfA, its homolog, may be a helper protein for the monooxygenase, CypX.

### 2.10. Conversion of Demethylsterigmatocystin (DMST) to Sterigmatocystin (ST) and Dihydrodemethylsterigmatocystin (DHDMST) to Dihydrosterigmatocystin (DHST)

Two *O*-methyltransferases, (I and II), are confirmed to be involved in aflatoxin biosynthesis [143]. *O*-methyltransferase I catalyzes the transfer of the methyl from *S*-adenosylmethionine (SAM) to the hydroxyls of DMST and DHDMST to produce ST and DHST, respectively. This 43-kDa enzyme was purified from *A. parasiticus* and characterized [144,145]. The corresponding gene, *dmtA*, was isolated from *A. parasiticus* based on a partial amino acid sequence of the purified enzyme [146]. Yu *et al*. [123] concurrently isolated the same gene but named it *aflO* (*omtB*) (for *O*-methyltransferase B) from *A. parasiticus*, *A. flavus* and *A. sojae*. The predicted *dmtA*-encoded protein contains a consensus SAM-binding motif [146]. The *aflO* (*omtB*) homolog in *A. nidulans* was identified as *stcP* this gene is required for the conversion of DMST to ST in *A. nidulans* as shown by gene disruption [147]. 

### 2.11. Conversion of Sterigmatocystin (ST) to *O*-Methylsterigmatocystin (OMST) and Dihydrosterigmatocystin (DHST) to Dihydro-*O*-methylsterigmatocystin (DHOMST)

The gene for *O*-methyltransferase required for the conversion of ST to OMST and DHST to DHOMST was first cloned [148] from *A. parasiticus* by reverse genetics using antibodies raised against the purified *A. parasiticus O*-methyltransferase A [78]. This gene was initially named *omt-1*, then *omtA* and finally renamed *aflP* (*omtA*) [148]. The recombinant enzyme was expressed in *E. coli* and its activity to convert ST to OMST was demonstrated by substrate feeding studies [148]. *O*-methyltransferase A has strict substrate-specificity and cannot methylate DMST or DHDMST. Thus, the *O*-methyltransferases A encoded by *aflP* (*omtA*) is the enzyme responsible for the conversion of ST to OMST and DHST to DHOMST. The genomic DNA sequence of this gene (*omtA*) was cloned from *A. parasiticus* and *A. flavus* [149]. This *aflP* (*omtA*) gene homologue was also detected in other aflatoxigenic and non-aflatoxigenic *Aspergillus* species [150]. The absence of the *aflP* orthologue in *A. nidulans* is the reason that *A. nidulans* produces ST as the end product instead of aflatoxins.

### 2.12. Conversion of *O*-Methylsterigmatocystin (OMST) to Aflatoxin B_1_ (AFB_1_) and Aflatoxin G_1_ (AFG_1_) and Dihydro-*O*-methylsterigmatocystin (DHOMST) to Aflatoxin B_2_ (AFB_2_) and Aflatoxin G_2_ (AFG_2_)

Based on feeding experiments, the relationship between B-group and G-group aflatoxin formation was proposed [151]. A P-450 monooxygenase gene in *A. flavus* named *ord-1* was shown to be necessory for this reaction [152,153]. This P-450 monooxygenase gene, *aflQ* (*ordA*), was cloned in *A. parasiticus* and demonstrated in a yeast system that it is involved in the conversion of OMST to AFB_1_/AFG_1_, and DHOMST to AFB_2_/AFG_2_ [154]. Whether *aflQ* (*ordA*) gene product, OrdA, catalyzes two successive monooxygenase reactions in the later steps of aflatoxin biosynthesis is not clear. Studies [154] suggested that additional enzyme(s) is required for the synthesis of G-group aflatoxins. After the cloning and characterization of the *cypA* gene, it is clear that *cypA* encoded a cytochrome P450 monooxygenase for the formation of G-group aflatoxins [155]. Most recently, the *nadA* gene, which was shown, by gene profiling studies using microarray, to be a member of the aflatoxin gene cluster [156,157] rather than belonging to the adjoining sugar utilization cluster as originally proposed [158], was found to play a role in AFG_1_/AFG_2_ formation. Yabe’s group recently disrupted the *nadA* gene and reported that NadA is a cytosolic enzyme for the conversion from a new aflatoxin intermediate named NADA, which is between OMST and AFG_1_, to AFG_1_ [159]. The *aflE* (*norA*) gene was initially believed to be involved in the conversion of NOR due to certain degree of sequence similarity to the *aflD* (*nor-1*) gene [108]. However, recent studies support the hypothesis that the *aflE* (*norA*) is involved in the final two steps in AFB_1_ formation [80]. In the same report, the transcript, *hypB*, a homolog of *hypC*, may be involved in one of the oxidation steps in the conversion of OMST to aflatoxins. *A. flavus* produces only AFB_1_ and AFB_2_, whereas *A. parasiticus* produces all four major aflatoxins, AFB_1_, AFB_2_, AFG_1_, and AFG_2_. Coincidently, only the G-group aflatoxin producer, *A. parasiticus*, has intact *nadA* and *norB* genes. Preliminary data suggests that *norB* encodes another enzyme predominantly involved in AFG_1_/AFG_2_ formation [160].

### 2.13. Aflatoxins M_1_ and M_2_

The aflatoxin M_1_ and M_2 _are mammalian bioconversion products of AFB_1 _and AFB_2_ respectively and are originally isolated and identified from bovine milk [161,162,163,164,165]. After entering the mammaliam body (human or animals), aflatoxins are metabolized by the liver cytochrome P450 enzymes to a reactive epoxide intermediate which becomes more carcinogenic, or be hydroxylated and become the less harmful aflatoxins M_1_ and M_2_. However, recent studies by feeding with aspertoxin (12c-hydroxy-OMST) [166] indicated that *A. parasiticus* produces the minor aflatoxins M_1_ (AFM_1_), M_2_ (AFM_2_), GM_1_ (AFGM_1_), and GM_2_ (AFGM_2_), as well as the major aflatoxins B_1_ (AFB_1_), B_2_ (AFB_2_), G_1_ (AFG_1_), and G_2_ (AFG_2_). It demonstrated that aspertoxin is a precursor of AFM_1_ and AFGM_1_. Feeding of the same fungus with *O*-methylsterigmatocystin (OMST), AFM_1_ and AFGM_1_ were formed together with AFB_1_ and AFG_1_; feeding with dihydro-OMST (DHOMST), AFM_2_ and AFGM_2_ were formed together with AFB_2_ and AFG_2_. It showed that the enzyme OrdA catalyzes both 12c-hydroxylation reaction from OMST to aspertoxin and the successive reaction from aspertoxin to AFM_1_ and the AFB_1_ is not a precursor of AFM_1_.

## 3. Genetic Regulation of Aflatoxin Biosynthesis

The aflatoxin pathway genes organized in cluster in the genome of *A. flavus* and *A. parasiticus* [67,81,87,167] are expressed concurrently. The positive-acting regulatory gene, *aflR*, is located in the middle of the gene cluster. Adjacent to *aflR* a divergently transcribed gene, *aflS* (*aflJ*), was found to be involved in the regulation of transcription [71,168]. Other physically unrelated genes, such as *laeA* and *veA*, have been shown to exhibit a “global” regulatory role on aflatoxin biosynthesis [169,170,171,172]. 

### 3.1. Genetic Control by aflR Gene, Encoding the Pathway-Specific Transcription Factor, AflR

A 47 kDa sequence-specific zinc-finger DNA-binding protein (AflR), encoded by the *aflR* gene, is required for transcriptional activation of most, if not all, the structural genes of the aflatoxin gene cluster [72,74,173,174,175,176,177,178,179,180]. Like other Gal4-type regulatory proteins that bind to palindromic sequences, functional AflR probably binds as a dimer. It binds to the palindromic sequence 5'-TCGN5CGR-3' in the promoter regions of the structural genes [77,181]. The AflR-binding motifs are found to be located from −80 to −600 positions, with the majority at the −100 to −200 positions, relative to the translation start site. AflR binds, in some cases, to a deviated sequence rather than the typical motif such as in the case of *aflG* (*avnA*). When there is more than one binding motif, only one of them is the preferred binding site such as in the case of *aflC* (*pksA*) [77,181]. The more upstream motif is found to belong to another gene for turning on the expression of *hypC*. Deletion of *aflR* in *A. parasiticus* abolishes the expression of other aflatoxin pathway genes [182]. Overexpression of *aflR* in *A. flavus* up-regulates aflatoxin pathway gene transcription and aflatoxin accumulation [176] in a fashion similar to that reported for *A. parasiticus* [173]. These results demonstrate that AflR is specifically involved in the regulation of aflatoxin biosynthesis. Indeed, all 23 upregulated genes, identified by transcription profiling using DNA microarray assays comparing wild-type and *aflR-*deleted *A. parasiticus* strains, have the consensus AflR binding motif in their promoter regions [156,168,183,184].

### 3.2. Genetic Control by aflS (aflJ) Gene, Encoding a Putative Transcriptional Co-activator, AflS

The *aflS* (*aflJ*) gene [168,185], bidirectionally transcribed from *aflR*, although not demonstrating significant homology with any other encoded proteins found in databases, is necessary for aflatoxin formation. The *aflS* and *aflR* share a 737 bp intergenic region. In the *A. parasiticus aflR* transformants, the production of aflatoxin pathway intermediates was significantly enhanced in transformants that contained an additional *aflR* plus *aflS* [173]. Quantitative PCR showed that in the *aflS* knockout mutants, the lack of *aflS* transcript is associated with 5- to 20-fold reduction of expression of some aflatoxin pathway genes such as *aflC* (*pksA*), *aflD* (*nor-1*), *aflM* (*ver-1*), and *aflP* (*omtA*). The mutants lost the ability to synthesize aflatoxin intermediates and no aflatoxins were produced [168]. However, deletion of *aflS* (*aflJ*) did not have a discernible effect on *aflR* transcription, and vice versa. Du *et al*. [186] showed that overexpression of *A. flavus aflS* (*aflJ*) did not result in elevated transcription of *aflM* (*ver-1*), *aflP* (*omtA*), or *aflR*, but it appears to have some effect on *aflC* (*pksA*), *aflD* (*nor-1*), *aflA* (*fas*-*1*), and *aflB* (*fas-2*) [186], which are required for the biosynthesis of the early aflatoxin pathway intermediates. The mechanism(s) by which *aflS* modulates transcription of these pathway genes in concert with *aflR* is under investigation.

### 3.3. Genetic Control on Secondary Metabolism by *laeA* Gene, Encoding a Global Regulator, LaeA

The novel global regulatory gene, *laeA* (for lack of *aflR* expression), was first identified from *A. nidulans* [169]. This gene is well conserved in fungi as shown by its presence in the genomes of all fungi so far sequenced. LaeA is a nuclear protein which contains an *S*-adenosylmethionine (SAM) binding motif and activates transcription of several other secondary metabolism gene clusters in addition to the AF cluster. Examples include the sterigmatocystin and penicillin clusters in *A. nidulans*, the gliotoxin cluster in *A. fumigatus*, and aflatoxin cluster in *A. flavus* [169,187]. It also regulates genes required for virulence of *A. fumigatus* [188]. Perrin *et al*. [170] carried out a whole-genome comparison of the transcriptional profiles of wild-type and *laeA*-deleted *A. fumigatus* strains and found that LaeA positively controls the expression of 20% to 40% of major classes of secondary metabolite biosynthesis genes. It also regulates some genes not associated with secondary metabolite clusters. Similar results were confirmed in gene expression profiling in *A. flavus* using microarrays to study the genetic mechanism of sclerotium formation. The exact mechanism of how LaeA regulates secondary metabolism gene clusters is not yet known. Interestingly, when an unrelated gene such as *argB* was placed within the boundary of the ST gene cluster, it was co-regulated with other genes in the cluster. But, when a gene in the cluster, such as *aflR* was placed elsewhere in the genome, its regulation was not affected by LaeA [189]. One proposed regulatory mechanism is that LaeA differentially methylates histone protein and it alters the chromatin structure for gene expression. Unlike the mentioned signaling factors, the primary role of LaeA is to regulate metabolic gene clusters, not sporulation, based on the obesevation that *laeA*-deleted strains produced conidia equivalent to wild-type level [169]. Most recent analyses of nonaflatoxigenic *A. parasiticus sec*- (for secondary metabolism negative) variants generated through serial transfer of mycelia of the *sec+* parents show that *laeA* was expressed in both *sec+* and *sec*- strains [190]. This result suggests that LaeA only exerts its effect on aflatoxin biosynthesis at a certain level and is independent of other regulatory pathways that are involved in fungal development.

### 3.4. Genetic Control on Fungal Development and Mycotoxin Formation by *veA* Gene, Encoding a Regulator, VeA

The *veA* gene in *A. nidulans* [191] is a gene initially found to be crucial for light-dependent conidiation. A comparison of the light effect on sterigmatocystin production by *A. nidulans veA+* and *veA1* strains showed that both strains produced sterigmatocystin but the highest amount was produced by the *veA*+ strain grown in darkness. However, *veA*-deleted *A. flavus* and *A. parasiticus* strains completely lost the ability to produce aflatoxin regardless of the illumination conditions [192,193]. Under normal growth conditions, some *A. flavus* and all *A. parasiticus* strains produce conidia in both dark and light conditions. Stinnett *et al*. [193] showed that VeA contains a bipartite nuclear localization signal (NLS) motif and its migration to the nucleus is light-dependent and requires the importin α carrier protein. In the dark VeA is located mainly in the nucleus; under light it is located both in cytoplasm and nucleus. VeA has no recognizable DNA-binding seuqences and likely exerts its effect on sterigmatosyctin and aflatoxin production through protein-protein interactions with other regulatory factors. Post-translational modifications such as phosphylation and dephosphorylation may modulate its activity. Lack of VeA production in the *veA*-deleted *A. flavus* and *A. parasiticus* strains consequently abolishes aflatoxin production because a threshold concentration of nuclear VeA might be necessary to initiate aflatoxin biosynthesis.

## 4. Factors that Affect Aflatoxin Biosynthesis

Many biotic and abiotic environmental factors influence aflatoxin biosynthesis [68,194,195,196,197] including nutritional factors such as carbon and nitrogen source; environmental effects such as water activity and temperature; physiological conditions such as pH [7] and bioreactive agents [198,199]. These studies would offer promise of devising control strategies to shut down aflatoxin production in aflatoxigenic *A. flavus* species through manipulations of environmental conditions for the fungal response to these factors.

### 4.1. Carbon

Carbon, nitrogen, amino acid, lipid, trace elements and other nutritional factors have long been observed to affect aflatoxin production [198,200,201]. The best-known nutritional factors affecting aflatoxin biosynthesis are carbon and nitrogen sources [202,203,204]. The relationship of carbon source and aflatoxin formation has been well established. Simple sugars such as glucose, sucrose, fructose, and maltose support aflatoxin formation, while peptone, sorbose, or lactose does not [198,205]. Woloshuk *et al*. reported the connection between alpha amylase activity and aflatoxin production in *A. flavus* [206]. Yu *et al*. identified a gene cluster related to sugar utilization in *A. parasiticus* next to the aflatoxin gene cluster [158]. A close physical linkage between the two gene clusters could point to a relationship between the clusters in the processing of carbohydrates leading to the induction of aflatoxin biosynthesis. Lipid substrate is a good carbon source to support aflatoxin production [207,208,209]. A lipase gene, *lipA*, was cloned in *A. parasiticus* and *A. flavus* [210]. The expression of *lipA* and subsequent aflatoxin production are induced by lipid substrate. The addition of 0.5% soybean oil to non-aflatoxin-conducive peptone medium induces lipase gene expression and leads to aflatoxin formation [210]. However, the molecular mechanism by which a carbon source is involved in the regulation of aflatoxin pathway gene expression is to be further investigated. 

### 4.2. Nitrogen

Nitrogen is closely linked to aflatoxin production [198]. Asparagine, aspartate, alanine, ammonium nitrate, ammonium nitrite, ammonium sulfate, glutamate, glutamine, and proline containing media support aflatoxin production; while sodium nitrate and sodium nitrite containing media do not [211,212,213]. It was also suggested that nitrate represses averufin and aflatoxin formation [214,215]. Nitrate was reported to have a suppressive effect on aflatoxin production, and overexpression of *aflR* gene by additional copies of *aflR* overcomes the negative regulatory effect on aflatoxin pathway gene transcription [173]. Nitrogen utilization genes and a nitrogen regulator gene, *areA* from *A. parasiticus* were cloned [216,217]. In the intergenic region between *aflR* and *aflS* (*aflJ*), several AreA binding motifs have been identified [216,217]. The AreA binding could prevent AflR binding. Certain amino acids can have different effects on aflatoxin production. Recent studies show that tryptophan inhibits aflatoxin formation while tyrosine enhances aflatoxin production in *A. flavus* [183]. 

### 4.3. Temperature

Aflatoxin formation is directly affected by temperature [218,219,220,221]. Optimal aflatoxin production is observed at temperatures near 30 °C (28 °C to 35 °C) [221]. When temperature increases to above 36 °C, aflatoxin production is nearly completely inhibited. Genome wide gene profiling using microarray and RT-PCR verification [221] indicated that high temperature is associated with a decrease in the expression of the aflatoxin pathway genes. RT-PCR detected ample amount of transcripts of both regulatory genes *aflR* and *aflS* (*aflJ*) [221]. So it was hypothesized that temperature may affect the activity of AflR or some other unknown regulatory element. High resolution studies using next generation sequencing technologies on aflatoxin pathway gene expression in response to temperature clearly showed that temperature affects the level of *aflR* and *aflS* transcripts [222]. High temperature affects *aflS* more than *aflR*. Change in the ratio of aflS to aflR renders *aflR* unfunctional for transcription activation.

### 4.4. Water Activity

Severe aflatoxin outbreaks in corn have been documented to occur under hot weather and drought conditions [223,224]. The mechanism of *A. flavus* infestation in corn under these conditions is not well understood. The possible scenarios may include a combination of these factors: (a) the plant defense mechanism is weakened under water stress conditions; (b) higher insect feeding and associated injuries to plant tissues, thus providing entry opportunities for fungal invasion; and (c) more fungal spores dispersed into the air under dry climate. 

### 4.5. Culture *pH*

Aflatoxin biosynthesis in *A. flavus* occurs in acidic media, but is inhibited in alkaline media [7]. The *pacC* gene is a major transcriptional regulatory factor for pH homeostasis [225]. In the *aflR* promoter region, at least one PacC binding site has been identified [77,181]. The presence of a putative PacC-binding site close to the *aflR* transcription start site may play some role in pH regulation on aflatoxin production [225,226]. In the non-aflatoxin-conducive peptone medium, this site was shown to be inhibitory to aflatoxin formation [7]. The regulatory mechanism might be due to the binding of *pacC* to this site at alkaline conditions to repress the transcription of acid-expressed gene *aflR* and thus aflatoxin formation [227,228]. The PacC and AreA [217] binding sites in the *aflR-aflS* (*aflJ*) intergenic region suggest that gene expression is regulated by environmental signals (pH and nitrate). 

### 4.6. Developmental Stage

Sporulation and sclerotial formation are associated with secondary metabolism [6,8,229,230]. Spore formation and secondary metabolite formation occur at about the same time [85,229]. Some mutants that are deficient in sporulation are unable to produce aflatoxins [68] and some compounds that inhibit sporulation in *A. parasiticus* also inhibit aflatoxin formation [231]. The aflatoxin-producing ability was gradually decreased in response to a series subculturing. The changes in aflatoxin production ability were accompanied by marked morphological changes [232]. 

Moreover, chemicals that inhibit polyamine biosynthesis in *A. parasiticus* and *A. nidulans* inhibit both sporulation and aflatoxin/ST biosynthesis [233]. A more recent finding reveals that the regulation of sporulation and ST production is through a shared G-protein mediated signaling pathway in *A. nidulans* [229,234]. Mutations in *A. nidulans flbA* and *fadA* genes, early acting members of a G-protein signal transduction pathway, result in loss of ST gene expression, ST production, and sporulation [179,229,235]. The regulation is partially mediated through protein kinase A [179,235]. This G-protein signaling pathway involving FadA in the regulation of aflatoxin production also exists in *A. parasiticus* and *A. flavus* [229]. 

### 4.7. Oxidative Stress

Oxidative stress and aflatoxin biosynthesis are related in *A. parasiticus* [236,237,238,239]. Oxidative stress induces aflatoxin formation in *A. parasiticus* [240]. Treatment of *A. flavus* with tert-butyl hydroperoxide, or gallic acid induced significant increases in aflatoxin production [238]. Similar treatment of *A. parasiticus* also induced aflatoxin production [239,241]. Hydrolysable tannins significantly inhibit aflatoxin biosynthesis, with the main anti-aflatoxigenic constituents in these tannins being gallic acid [237]. Gallic acid reduces expression of structural genes within the aflatoxin biosynthetic cluster, but surprisingly not the aflatoxin pathway gene regulator, *aflR*. It appears that gallic acid disrupts signal transduction pathway(s) for aflatoxigenesis. When certain phenolics or other antioxidants, such as ascorbic acid, are added to oxidatively stressed *A. flavus*, aflatoxin production significantly declines, with no effect on fungal growth [238]. Caffeic acid is another antioxidant that inhibits aflatoxigenesis. Microarray analysis of *A. flavus* treated with caffeic acid identified a gene, named *ahpC2*,an alkyl hydroperoxide reductase that is potentially involved in quelling the signal for aflatoxin production. However, no notable effect on expression of *laeA*, a gene encoding a global regulator for secondary metabolism in *Aspergillus* [169] was observed when under caffeic acid treatment. 

### 4.8. Plant Metabolites

Plant metabolites play some role on aflatoxin formation [242,243,244,245]. Wright *et al*. reported that, at certain conditions, *n*-decyl aldehyde reduces not only fungal growth of *A. parasiticus* but also aflatoxin production by over 95% compared with control [246]. Octanal reduces fungal growth by 60%, however, it increases aflatoxin production by 500%, while hexanal reduces fungal growth by 50%, but it has no effect on aflatoxin production. The 13(*S*)-hydroperoxide derivative of linoleic acid, the reaction product of lipoxygenase (encoded by L2 LOX gene from maize), is reported to reduce aflatoxin production [247]. Therefore, besides routine detection and screening, management of aflatoxin contamination in commodities must include pre-harvest as well as post-harvest control measures.

## 5. Future Perspective in Control of Aflatoxin Contamination

The economic implications of aflatoxins and their potential health threat to humans have clearly shown a need to eliminate or at least minimize its contamination in food and feedstuff. Efforts include monitoring, managing and controlling their levels in agricultural products from farm to market and from preharvest to post-harvest.

### 5.1. Detection and Screening

Surveillance programs have been established to reduce the risk of aflatoxin consumption in humans and animals. Analytical testing methods for rapid detection of a large number of samples of food stuffs have been developed [83]. Current analytical techniques for accurate characterization and quantitation of aflatoxins include thin layer chromatography (TLC), high pressure liquid chromatography (HPLC) and gas chromatography (GC). Rapid immuno-assay (RIA) and serum assay (ELISA) formats based on sera developed for major aflatoxins [83,248,249] are also available for rapid detection of aflatoxins at levels as low as one tenth of a nanogram per milliliter [250]. A dozen commercial test kits have been developed for field-testing of various agricultural commodities.

### 5.2. Preharvest Control

Aflatoxin contamination can be reduced somewhat by appropriate cultural practices such as irrigation, control of insect pests etc prior to harvest. In some cases, changes in cultural practices to minimize aflatoxin contamination are not feasible. For example, inclusion of extra irrigation regimes in desert cotton fields is not feasible because it would add significant cost to the grower even if they were available. However, effective control of aflatoxin contamination is expected to be dependent on a detailed understanding of the physiological and environmental factors that affect aflatoxin biosynthesis, the biology and ecology of the fungus, and the parameters of the host plant-fungal interactions. Efforts are underway to study these parameters, primarily by functional genomics approaches [251,252].

### 5.3. Postharvest Control

Aflatoxin contamination during storage after harvest is prevalent in most tropical countries due to hot, wet climates coupled with sub-adequate methods of harvesting, handling and storage practices, which often lead to severe fungal growth and thus contamination of food and feed [253,254]. Significant emphasis has been placed on improving storage conditions that are less favorable for fungal growth. Detoxification of aflatoxin contaminated grains to reduce aflatoxin to an acceptable level is another strategy in corn and peanuts [255]. 

### 5.4. Biological Control

Application of nonaflatoxigenic, biocompetitive, native *A. flavus* strains to outcompete toxigenic isolates in the fields has been effective in significantly reducing aflatoxins contamination [256]. Biocompetition may be most feasible in crops such as cotton where resistance against fungal infections is not available due to limited genetic diversity in the cotton germplasm. In the long term, however, significant control of the aflatoxin problem will likely be linked to introduction of resistant germplasms, which are resistant either to fungal invasion or toxin production or both. Naturally resistant germplasms are available and the identification of specific biochemical factors linked to resistance against *A. flavus* will assist in enhancing the observed resistance levels in the existing germplasms. Identification of a novel pool of germplasm that demonstrates the desired characteristics is also critical to the success of the current marker-assistance breeding programs. However, because the aflatoxin contamination is a complex problem and a combination of approaches will be required to control the preharvest contamination.

### 5.5. Breeding for Commercial Crops that Are Resistant to Fungal Growth and/or Aflatoxin Formation

The most economic and effective strategy for reducing and eliminating aflatocin contamination is to breed commercial crop varieties that show resistance to fungal infection and/or aflatoxin formation. Significant efforts are made in plant breeding programs in identifying resistance or tolerance to aflatoxin contamination in preharvest crops. Unfortunately, no highly resistant varieties or germplasm lines have been identified for the major crops such as corn, cotton, and peanut. Some low to medium resistant lines in corn are under testing and development [257,258]. Progress has been made in identifying genes in corn that shows resistance to aflatoxin producing fungus [259].

## 6. Conclusions

Scientists worldwide have extensively studied biosynthesis of aflatoxins for more than 50 years. The more we learn concerning the mechanisms of aflatoxin biosynthesis, the more we need to investigate its regulatory mechanisms. Regulation of aflatoxin gene expression occurs at multiple levels and by multiple regulatory components. There are genetic factors, biotic and abiotic elements that affect aflatoxin formation. Studies revealed the functions of the enzymes involved in each of the steps of aflatoxin biosynthesis, the genes encoding those enzymes, and the regulatory mechanisms of aflatoxin formation. With the rapid progress in fungal genomics, we will master a vast amount of new information on gene function, genetic regulation and signal transduction within this fungal system, as well as its interactions with the environment. It is now time to supplant the classic gene cloning strategy with the cutting-edge whole genome approach including the Next Generation Sequencing technologies. The genetic and genomic resources will significantly enhance our understanding of the mechanisms of aflatoxin production, pathogenicity of the fungus, and crop-fungus interactions. Better understanding of the mechanisms of gene regulation on aflatoxin biosynthesis will help us to identify natural inhibitors of fungal growth and aflatoxin formation. Eventually, we will be able to design effective and novel strategies to eliminate aflatoxin contamination for a safer, nutritious and sustainable food and feed supply.
